# Resident perceptions of the impact of duty hour restrictions on resident-attending interactions: an exploratory study

**DOI:** 10.1186/s12909-017-0963-7

**Published:** 2017-07-18

**Authors:** Kristen A. Gerjevic, Marcy E. Rosenbaum, Manish Suneja

**Affiliations:** 1 Department of Obstetrics and Gynecology, Dartmouth Hitchcock Medical Center, Lebanon, NH USA; 2 Department of Family Medicine, University of Iowa Carver College of Medicine, Iowa City, IA 52242 USA; 3 Department of Internal Medicine, University of Iowa Carver College of Medicine, Iowa City, IA USA; 4 Department of Internal Medicine, University of Iowa Hospitals and Clinics, Iowa City, IA 52242 USA

**Keywords:** Duty hour restrictions, Graduate medical education, Hidden curriculum, Resident perceptions

## Abstract

**Background:**

The institution of duty hour reforms by the Accreditation Council for Graduate Medical Education in 2003 has created a learning environment where residents are consistently looking for input from attending physicians with regards to balancing duty hour regulations and providing quality patient care. There is a paucity of literature regarding resident perceptions of attending physician actions or attitudes towards work hour restrictions. The purpose of this study was to identify attending physician behaviors that residents perceived as supportive or unsupportive of their compliance with duty hour regulations.

**Methods:**

Focus group interviews were conducted with residents exploring their perceptions of how duty hour regulations impact their interactions with attending physicians. Qualitative analysis identified key themes in residents’ experiences interacting with faculty in regard to duty hour regulations. Forty residents from five departments in two hospital systems participated.

**Results:**

Discussion of these interactions highlighted that attending physicians demonstrate behaviors that explicitly or implicitly either lend their support and understanding of residents’ need to comply with these regulations or imply a lack of support and understanding. Three major themes that contributed to the ease or difficulty in addressing duty hour regulations included attending physicians’ explicit communication of expectations, implicit non-verbal and verbal cues and the program’s organizational culture.

**Conclusions:**

Resident physicians’ perception of attending physicians’ explicit and implicit communication and residency programs organization culture has an impact on residents’ experience with duty hour restrictions. Residency faculty and programs could benefit from explicitly addressing and supporting the challenges that residents perceive in complying with duty hour restrictions.

## Background

In 2003 the Accreditation Council for Graduate Medical Education (ACGME) instituted duty hour reform in response to congressional pressure for national regulation. The major element of this reform included the limitation of duty hours to 80 h per week averaged over a 4-week period [1]. Many opinions have been published questioning the quality of training and types of residents entering post-graduate training today [2]. There are concerns that residents have become ‘shift-workers’ and are given less autonomy due to decreased training time across all residency specialties [3]. In a 2010 study, attending physicians felt that duty hour restricted residents demonstrated a lower baseline work ethic and less developed technical skill set, decision-making ability and sense of patient ownership [4]. There have also been concerns raised that shorter duty hours may erode the professional allegiance of these residents to patients [5]. Among surgical program directors there is a concern that their residents are not well-equipped to practice autonomously [6]. A recent study has raised further questions about the validity of the current surgical resident duty-hour policies as it found no difference in patient outcomes, resident education and resident well-being when compared to a relaxed duty-hour policy [7].

Residents have also felt the trade off between work hours and training. One study explored the ethical dilemma residents face in balancing duty hour restrictions (DHR) with patient care and revealed that a significant number of residents feel compelled to exceed DHR and report those hours falsely. Of note, primary reasons identified for non-compliance and underreporting of hours by residents included concerns about the impact on patient care, educational experiences, and meeting expectations of both supervising senior residents and faculty [8, 9]. Another recent study assessed professionalism under DHR using direct observation. This study suggested that residents under-reporting of duty hours was not simply an issue of telling the truth or lying. Rather it involved a complex thought process where residents weighed multiple factors including the importance of a compliant program, their own reputation, and an inability to recall their hours at the end of a reporting period. Motivation to stay past duty hours were generally attributed to being unable to complete work in the time allotted and concerns about diminishing the quality of patient care if they left on time [10].

These opinions and interactions have created a learning environment in which residents are consistently seeking input from the attending physician with regards to balancing DHR and providing quality patient care [8–10]. While there have been many published opinions about the value of DHR, there is a paucity of literature regarding resident perceptions of attending physician actions or attitudes towards work hour restrictions [11]. In the last 20 years the concept of the “hidden curriculum” has received much attention from the medical education community [12–14]. The premise of this hidden or informal curriculum is that behaviors such as professional behavior are learned not only in formal educational sessions but also in learners’ day-to-day interactions with faculty, residents, staff and patients in the context of clinical care. Through these interactions, implicit messages are conveyed about what is and is not valued within the medical education program or institution. The goal of our research was to examine residents’ perspectives on how interactions between residents and supervising faculty related to residents’ work ethic and professional responsibilities have been influenced by DHR. Thus, this study aimed to explore the hidden and not-so- hidden curriculum that residents experience in relation to DHR.

## Methods

When assessing behavior trends and experiences, particularly about which little is known, it is common to use qualitive methods such as focus groups and surveys [15, 16]. This study analyzed resident perception of attending physician attitudes towards DHR using both qualitative and quantitive methods.

The project was approved by the Institutional Review Board in October 2012 and focus groups were started in December 2012. Participants included residents from a variety of specialties (Family Medicine, General Surgery, Pediatrics and Internal Medicine) from two different sites – a large University based teaching hospital and a community based teaching hospital, both located in the Midwestern United States.

Program coordinators in each program helped identify convenient meeting times for resident focus groups and forwarded informational recruitment emails to all residents in each program asking for their voluntary participation. Two of the focus groups were conducted during reserved educational time and the other three during times set aside by program directors. Program directors were not present for any of the sessions and an assistant program coordinator was present for one of the sessions. The number of resident participants ranged from 4 to 20 in each group averaging 8 participants per group.

We used a multi-method approach including open-ended and likert type individual survey questions and focus group discussion to identify the range of resident perspectives of faculty attitudes and behaviors related to DHR. Survey and focus group questions were developed based on a review of resident DHR issues in the literature and the specific focus of this research project. The surveys and questions were piloted with 3 residents and refined prior to initiation of the focus groups. In order to capture each individual resident’s perspective, each participant was given a survey to complete at the beginning of the focus group which contained open ended questions about the impact of work hours on their interactions with faculty. In addition, they answered 4 likert-type questions about their experiences with faculty attitudes toward DHR (1 = Never – 5 = All the Time). (See Tables 1 and 2). Respondents were identified only by their residency program and post-graduate year. No names or other personal information was recorded to maintain anonymity. All but one of the focus groups were conducted by two of the authors, one of whom had extensive training in qualitative interview methods (MR) and coached the first author (KG) in these methods (one group had only one author facilitate). Focus groups were audio recorded and notes were taken in order to get an accurate account of the dialogue. During the focus group, participants were asked to discuss their responses to the open ended items on the survey specifically focusing on interactions between attending physicians and duty hour restrictions. Focus group discussion of their survey responses allowed for issues to be discussed in more depth and for participants to react to one another’s statements, thus supplementing and providing further explanation of the range of perspectives noted in the survey responses. Each focus group lasted roughly 1 hour.

**Table 1:** Open ended survey questions

1. How do work hour restrictions impact the interactions between attending physicians and resident physicians? Please describe both positive and negative impacts.
2. Attending physicians may be concerned that duty hour restrictions have compromised resident professionalism. In your experience, what concerns do you think they have?
3. Do you feel your evaluations will be affected depending on how well you comply with duty hours? If so, negatively or positively?

**Table 2:** Resident responses to questions about attending attitudes toward DHR

Survey question: To what extent have you experienced each of these attitudes or statements?	Never	Sometimes	Often
Under duty hours rules, residents may become shift workers regulating their work hours according to the clock instead of their patients’ needs	5^a^	19	14
When I was training, we worked much longer hours and were better physicians for it	0	15	23
Current resident emphasis on work-life balance isn’t congruent with being a good physician	7	26	5
Exceeding duty hours means that the resident is inefficient or doesn’t use his/her time effectively	1	24	11

^a^Number of residents giving each rating – 1 = Never, 2–3 = Sometimes, 4–5 = Often or All the Time

*N* = 2 did not answer the first three questions. *N* = 4 did not answer the last question

**Fig. 1:**
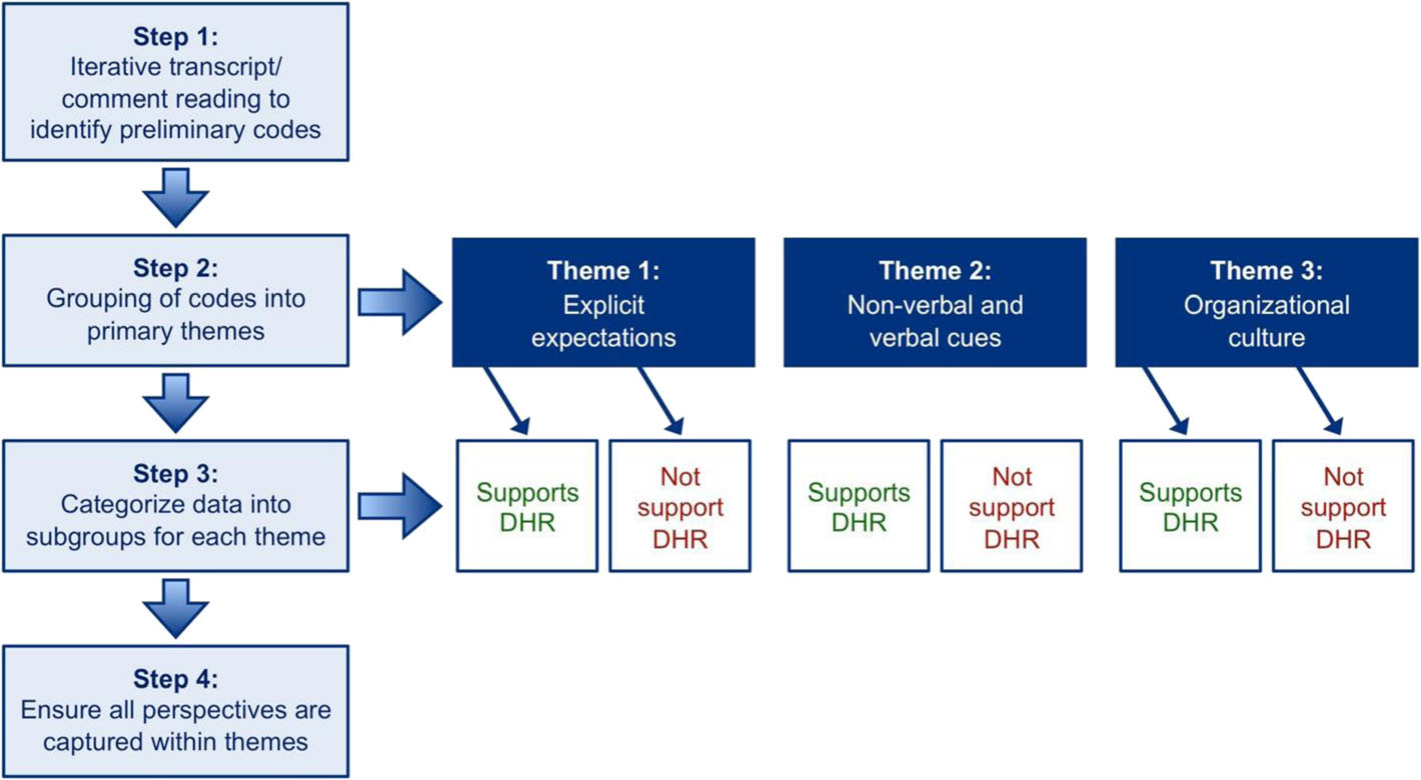
Thematic analysis process identifying resident perceptions of attending physicians’ expressing their attitudes toward DHR

Thematic analysis was utilized to identify the main ways in which residents perceived attending physicians’ expressing their attitudes toward DHR [15, 16]. The first two authors (KG and MR) closely read through all the survey comments and focus group transcripts to identify initial themes which provided the basis for a preliminary coding scheme. Each author then applied the coding scheme to a sample of comments and transcripts and compared their coding to resolve any conflicting interpretations or new codes that emerged from the analysis. The final coding scheme was subsequently applied to all the data from the surveys and focus group conversations using NVivo 8 software, which allowed for systematic searching and sorting of data [17]. To ensure quality and rigor of the data, all coded data and codes were subsequently reviewed by the third author (MS) to ensure that all data could be accounted for by the main themes. See Fig. 1 for a summary of the analysis process and main themes. Responses to likert questions were summarized by identifying the number of respondents for each rating category.

## Results

A convenience sample of forty residents participated in this study from four different disciplines (Family Medicine, Surgery, Pediatrics and Internal Medicine) in two hospital systems. Participants included 24 general surgery residents and 16 residents from non-surgical disciplines. Twenty five participants were in an academic residency program (Family Medicine and General Surgery), and 15 in a community based residency program (Pediatrics, Internal Medicine, General Surgery). Participant level included 8 PGY 1, 12 PGY 2, 12 PGY 3 and 8 PGY 4 or above.

Residents identified a range of interactions they have with faculty related to DHR. Discussion of these interactions highlighted that attending physicians demonstrate behaviors that explicitly or implicitly either lend their support and understanding of residents’ need to comply with DHR or imply a lack of support and understanding. In speaking with residents, three major themes emerged that contributed to ease or difficulty in addressing DHR issues: explicit expectations, implicit verbal and non-verbal cues, and organizational culture. Here we present each of these main themes, which represent the most salient aspects of how all participating residents perceived the impact of DHR of attending-resident interactions. Within each theme we explore the most consistent range of perspectives presented by participating residents.

### Explicit expectations

Explicit communication from attending physicians to residents was perceived as communicating expectations and either lack of support or support for resident DHR. Residents identified the ways in which some attending physicians would compare current training rules with their own training as communicating negative attitudes about DHR. They cited typical comments from these faculty as focusing on how much harder they had to work, how much more they learned and how current residents will not be prepared for the ‘real world’. As one resident noted:

“They seem to think we will be less well-trained than they were and less willing to work, trading work experience for lifestyle.”

Participating residents also noted faculty comments that were directed not at the quality of training but the quality of resident. Residents interpreted these comments as faculty perceiving residents as not wanting to work hard under DHR even though they have no choice but to comply. Some residents commented:

“Many [attending physicians] mention how they ‘used to do things’. We appear weak and coddled.”

“One subspecialist came and told us ‘You leave by 6, watch a movie and spend time with your family, take a bath, sleep 6 hours and then come back to work’.”

Related to these perceptions, some residents noted that reminding attending physicians of DHR can be a difficult task. Attending physicians are busy and may not be as familiar with residents’ schedules especially in larger institutions with many residents. There is an inherent conflict when a resident has to tell an attending that he/she has to leave in order to comply with DHR. Residents identified feeling they risk the perception that the resident is lazy and is not invested in the care of his/her patients or training. This could translate into poor evaluations, poor working relationships with superiors and a poor reputation among colleagues. For example, some residents said:

“If I appear anxious to remind physicians that I am "off shift" I think they will assume that I am lazy.”

“I would have difficulty discussing my concerns [exceeding DHR] and normally would just deal with it and hope for a better night tomorrow. I would be concerned about a bad evaluation from staff and other residents.”

In contrast, residents appreciated faculty who were explicit in their communication reinforcing DHR rules, which gave them confidence to make decisions about DHR. Residents reported three main ways in which faculty would communicate their support and understanding.

First, many residents noted that attending physicians “tell you to go home” and that allows residents to leave without concern of repercussions. Second, when faculty communicated awareness of residents’ schedules and duty hour rules, this was seen as reinforcing support for residents’ compliance with DHR.

“Mainly as an intern I remember rounding the next morning, seeing the attending. .. and he’s like, ‘Hey! Weren’t you on last night? It’s time for you to leave.’ And so that’s communication of ‘Good job! I recognize your effort, but we need to follow the rules’.”

Finally, residents reported that when faculty explicitly stated their support of DHR to house staff, it reinforced the importance of complying with DHR and allowed residents to not feel guilty about leaving the hospital.

“.. . one of our staff members who said this is a great thing that we went to these [DHR] and you know, openly showed support for them, which I think is encouraging to all of us because every once in a while you do feel like you know, oh, am I missing out on something because I’m not here as much as I used to be. ..”

Thus, when residents felt supported by their faculty and faculty explicitly stated their belief in DHR rules, residents were more comfortable telling their faculty that they need to leave to comply with DHR:

“I think attendings in our department are for the most part all supportive of the [DHR] so we don’t feel a lot of issues here. I mean they still have to be reminded to make sure they get people out on time, but you know, at least some people [faculty] I’ve talked to, they do believe the new work rules are better for patient care based on the research data.”

### Implicit verbal and non-verbal cues

Residents also identified both verbal and non-verbal actions by supervising faculty provided cues that they have to interpret to understand faculty acceptance of DHR. Some of these actions have made residents think twice about addressing DHR. A senior resident noted on his survey:

“[In response to me having to leave because of DHR], some sigh, roll their eyes or make subtle (or not-too-subtle) comments – like teenagers do. I think they are frustrated but know their hands are tied.”

Another said, “[On specialty service] sometimes we have to miss a day of the week to not violate hours. You have to tell the attending (and then you) get a lot of pen dropping.. .lots of pen throwing.”

On the other hand, some residents reported actions and cues perceived as supportive that enable residents to move through work faster and allowed them to leave the hospital in a time-sensitive fashion. These include re-organizing rounds, offering to help see patients, entering orders, or taking call. Many times the faculty member would verbally acknowledge the time constraint and then state his/her intent to efficiently move through rounds. When asked about supportive behaviors, residents identified:

“A few attendings will ask if you were on call and hurry with rounds and stay focused to get work done efficiently”.

“Many attendings will ‘pitch in’ and take first call if a resident is over hours.”

### Organizational cultures

The third major theme that impacts interactions between residents and faculty in relation to DHR is organizational culture. Scheduling, cultural values and experience shape the environment in which a resident is trained.

First, resident scheduling can determine the interactions a resident may have with supervising physicians in regards to DHR. For instance, residents noted that if a schedule does not take into consideration busy services or allow sufficient time for residents to complete their work (e.g. see patients, write orders and document, etc), the program is placing their residents in difficult situations with regards to DHR compliance. For example, some residents commented that they are doing more in less time:

“Faculty who do not have a lot to do with work hours recommend that I come in early to get my work done, but I can’t do that. They want me to be more efficient but no real suggestion on how to do that.”

“If you are supposed to see 15 patients in an hour, then you are seeing 1 person every 4 minutes. That is physically impossible.”

Second, cultural values play an important role in creating a comfortable or uncomfortable environment between residents and faculty. In situations where residents feel that faculty valuing of the DHR does not necessarily align with resident or program views, they will be more hesitant to approach attending physicians with their concerns. In fact, they may feel that they need to alter their actions in order to find a peaceful middle ground with faculty.

“I’m not going to be happy to tell them I have to take tomorrow off [because of DHR]. You have to act like you don’t agree with duty hours but have to comply. You have to portray yourself as a victim of duty hours.”

However, when the program schedules residents with those aforementioned obstacles in mind, residents do not find themselves needing to address DHR with supervising faculty. Residents noted the importance of explicitly educating residents about DHR rules and exceptions to allow them to take ownership of patient care.

“The way the schedule is set up, I mean, this was taken care of on the front end, so we are not running into an issue like this [exceeding DHR], but I mean, even if you do get into that situation, you are still given some of that autonomy too, if somebody is sick and you want to stay to take care of them, you can make that choice on your own and do that.. .”

There is less conflict when residents and faculty view DHR rules with a similar opinion, regardless of compliance. For example, if both groups are frustrated with DHR, residents will be more comfortable discussing DHR with faculty:

“[Attendings] realize that it’s not what we want. It’s the rules. We want our program to be strong and in good standing; therefore, we comply with the rules. But, me, as an individual resident, I don’t like it.”

Similarly, if both residents and faculty believe DHR is a good principle, the environment in which residents need to comply with DHR is perceived as a positive one:

“The majority of our staff is very supportive of [DHR]. I’d say 95%ish. I feel like they’re usually the ones who say, ‘You need to get out of here, you’ve been here too long. You have to come back tomorrow.’. .. The program director is very proactive and educates the staff over and over again.”

### Resident perceptions of faculty attitudes

Analysis of responses to Likert- type questions reinforced and provided additional insight on residents’ experiences interacting with supervising faculty in the context of DHR (See Table 2). Residents noted, either often (*n* = 23) or at least sometimes (*n* = 15), that the most common attitude from faculty was that when they were training they worked longer hours and were better physicians for it. Experience of the other attitudes toward DHR was much more variable, with residents noting that the perception that exceeding DHR was a reflection of resident efficiency as happening at least some times (*N* = 24) if not often (*N* = 11) and the perception of residents as shift workers driven by the clock rather than patient needs as being experienced sometimes (*N* = 19) or often (*N* = 14). Respondents less commonly identified negative attitudes toward work-life balance when asked to identify which group of attending physicians were perceived as most often displaying these attitudes, the majority of residents felt they were most common among faculty who had been practicing for 10 or more years (*N* = 27) and several commented that younger faculty trained under DHR had more positive attitudes and understanding towards adhering to these restrictions.

For both the narrative and quantitative data, while there appeared to be differences in resident perceptions between programs and disciplines, the small number of participants precludes drawing any significant conclusions of the impact of discipline or program location on these perceptions.

## Discussion

Even though it has been 10 years since the first ACGME DHR were implemented, residents still report concerns addressing DHR with attending physicians. Reasons for these concerns have been previously discussed in the literature and include attending perceptions of laziness, decreased work ethic and overall decreased quality of resident training [3, 4]. There is also evidence that attending physicians believe that DHR has adversely affected important aspects of residents’ patient care, education and professionalism [15]. For example, in one study attending physicians believed that residents’ accountability to patients and ability to place needs of patients and society above self-interests have worsened [18]. These findings, though concerning, should be interpreted with the understanding that most current attending physicians were trained in an era without limitations on duty hours. Residents’ requirement to comply with DHR potentially conflicts with attendings’ beliefs about placing the needs of patients and society above personal needs as a professional obligation of physicians [19, 20]. There is limited literature exploring attending behaviors that residents perceive as encouraging compliance with DHR. The current study has empirically investigated, from the perspective of residents, attendings’ behaviors and organization culture that encourage resident compliance with DHR. The purpose of this exploratory study was to identify the range, rather than prevalence, of perceptions that residents may have regarding attending attitudes and behaviors. As our results demonstrate, we were able to elicit both positive and negative perceptions regarding DHR from the residents with examples. These findings point to the way residents experience the hidden or “not-so-hidden” curriculum in relation to DHR [12–14].

This study has important implications for both faculty and programs in terms of supporting and encouraging residents’ compliance with DHR. At an individual faculty level clear communication of expectations regarding DHR with the residents helps encourage compliance with these regulations. Clearly stated expectations and a culture of collegiality among residents and faculty will build a comfortable, professional atmosphere which will improve compliance with DHR. Individual faculty should be aware of how their explicit and implicit communication impacts residents’ experience with duty hour restrictions. The receptiveness and approachability of the individual faculty to re-organize teaching rounds, help with notes or orders, see additional patients for the resident, and helping take call and address duty hour concerns could help improve compliance with DHR. On the other hand, either lack of responsiveness on the part of an individual attending faculty, which can be expressed as an absence of intervention to address duty hour compliance problems, or actions and statements critical of DHR may negatively affect compliance with these regulations.

Organizational and program culture is also an important component of residents’ comfort level with DHR. Well-developed clinical infrastructure that facilitates residents’ ability to complete their educational and clinical obligations within the allowed hours would help residents comply with DHR. It is imperative that programs and organizations invest in the infrastructure as well as support staff to address resident clinical load and efficiency. The program culture including working knowledge of DHR amongst the supervising faculty would be helpful in supporting residents’ compliance with DHR.

This study also found that conflict between residents and attending physicians decreased when the opinions regarding DHR aligned. For instance, if both residents and faculty did not agree with DHR principles, compliance notwithstanding, then residents felt there was less tension with faculty. However, in programs where residents felt like duty hours were necessary but faculty did not agree, tension was increased.

One limitation of this study is the small number of residency programs that participated and the limited numbers of resident participants within each of the focus groups. However, the number of programs and participants was appropriate for an exploratory study of this type seeking to identify the range of perspectives that residents have in regard to this issue [12, 13]. The multimethod approach used, combining open ended survey questions with focus groups, allowed for a maximum range of perspectives to be gathered and analyzed. It would be advantageous to expand this study to other programs at different institutions to see if the range of responses and main themes identified in this study are more generalizable. The small sample size also precluded drawing any substantial conclusion about the impact of specific discipline or location on resident perceptions. Thus, conducting a similar study with a larger sample would allow identification of differences in perceptions and experiences of residents at different levels and in different disciplines.

The purpose of this study was to identify attending physician behaviors that residents perceived as supportive or unsupportive of their compliance with DHR. However, it was beyond the scope of this study to directly correlate those behaviors with actual or reported work hours. Further studies may be warranted to analyze the implications for residency training and what potential strategies may exist to appropriately handle these types of situations. In addition studies specifically looking into the faculty perception of DHR and its actual impact on the residents’ compliance with DHR would be helpful in answering whether residents’ perceptions were in fact real or just perceived.

## Conclusion

In summary, this exploratory study has identified residents’ perceptions of some of the actions of attending physicians and residency organizational culture that impact residents’ experiences with DHR. This study provides some very specific actions that program directors can take to be more supportive of these regulations. What is striking is that while DHR rules are now far from new, attending physicians and residency programs appear to vary in the extent to which their actions, explicit and/or implicit, demonstrate support for resident compliance with these regulations. This study raises concerns that the “hidden curriculum” and biases regarding DHR continue to exist and manifest themselves in both implicit and explicit attending behaviors which subsequently may impact resident behaviors and choices about compliance. Creating a stronger relationship between supervising faculty and residents is important to residency programs especially in the context of DHR. It is important that faculty and programs are aware of the impact of this “not so hidden” curriculum related to DHR on the learning environment for residents and to adjust their approaches in order to build a good relationship with residents and encourage compliance with DHR.

## Abbreviations

ACGME: Accreditation Council for Graduate Medical Education (ACGME)
DHR: Duty hour restrictions

## References

[1] Philibert I, Friedmann P, Williams WT (2002). New requirements for resident duty hours. JAMA.

[2] Resident SM. Duty hours: are we focusing on the right metric? SGIM Forum. 2013:36(8).

[3] Coverdill JE, Carbonell AM, Cogbill TH (2011). Professional values, value conflicts, and assessments of the duty-hour restrictions after six years: a multi-institutional study of surgical faculty and residents. Am J Surg.

[4] Griner D, Menon RP, Kotwall CA, Clancy TV, Hope WW (2010). The eighty-hour workweek: surgical attendings' perspectives. J Surg Educ.

[5] Arora VM, Farnan JM, Humphrey HJ (2012). Professionalism in the era of duty hours: time for a shift change?. JAMA.

[6] Antiel RM, Van Arendonk KJ, Reed DA, Terhune KP, Tarpley JL, Porterfield JR, Hall DE, Joyce DL, Wightman SC, Horvath KD, Heller SF, Farley DR (2012). Surgical training, duty-hour restrictions, and implications for meeting the accreditation Council for Graduate Medical Education core competencies: views of surgical interns compared with program directors. Arch Surg.

[7] Bilimoria KY, Chung JW, Hedges LV (2016). National clusterrandomized trial of duty-hour flexibility in surgical training. N Engl J Med.

[8] Carpenter RO, Austin MT, Tarpley JL, Griffin MR, Lomis KD (2006). Work-hour restrictions as an ethical dilemma for residents. Am J Surg.

[9] Carpenter RO, Spooner J, Arbogast PG, Tarpley JL, Griffin MR, Lomis KD (2006). Work hours restrictions as an ethical dilemma for residents: a descriptive survey of violation types and frequency. Curr Surg.

[10] Szymczak JE, Brooks JV, Volpp KG, Bosk CL (2010). To leave or to lie? Are concerns about a shift-work mentality and eroding professionalism as a result of duty-hour rules justified?. Milbank Q.

[11] Rybock JD (2009). Residents' duty hours and professionalism. N Engl J Med.

[12] Hafferty FW (1998). Beyond curriculum reform: confronting medicine's hidden curriculum. Acad Med.

[13] Haidet P, Kelly A, Chou C (2005). Characterising the patient-centredness of hidden curriculum in medical schools. Acad Med.

[14] Glicken AD, Merenstein GB (2007). Addressing the hidden curriculum: understanding educator professionalism. Med Teach.

[15] Rice PR, Ezzy D (2000). Qualitative research methods: a health focus.

[16] Denzin NK, Lincoln YS (2000). Handbook of qualitative research.

[17] QSR International Pty Ltd. Nvivo Qualitative data analysis software version 8. Doncaster,Victoria, Australia; QSR International Pty Ltd, 2007.

[18] Reed DA, Levine RB, Miller RG (2007). Effect of residency duty-hour limits: views of key clinical faculty. Arch Intern Med.

[19] Larriviere D (2004). Duty hours vs professional ethics: ACGME rules create conflicts. Neurology.

[20] Smith LG (2005). Medical professionalism and the generation gap. Am J Med.

